# Crossmodal attention effects on brain responses to different stimulus classes

**DOI:** 10.1186/1471-2202-7-31

**Published:** 2006-04-11

**Authors:** Wido Nager, Kaija Estorf, Thomas F Münte

**Affiliations:** 1 Department of Neuropsychology, Otto-Von-Guericke Universität Magdeburg, 39106 Magdeburg, Germany; 2 Department of Neurology, Medizinische Hochschule Hannover, 30623 Hannover, Germany

## Abstract

**Background:**

Attending to a point in space in one modality may facilitate processing to information from the same region in another modality. The involvement of sensory-specific cortical areas in intramodal and crossmodal selective spatial attention can be assessed with event-related brain potentials (ERPs).

ERPs were recorded in two groups of young participants (each n = 11). Stimulus sequences comprised visual standard stimuli (p = 0.4, a white square), visual deviant stimuli (p = 0.05, a white square with small black rectangle) and visual novel stimuli (p = 0.05, different multicolored checkerboards) as well as auditory standard (p = 0.4, 800 Hz tone), deviant (p = 0.05, 900 Hz tone), and novel (p = 0.05, random combination of three sine-wave tones) stimuli, occurring in random order at locations 30 degrees left and right of a fixation point. The "auditory group" of participants attended either to the left or to the right speaker in order to respond to the infrequent auditory deviants at that location by a speeded button press. Visual stimuli were irrelevant for this group. The "visual group" had the analogue task for the visual modality. For these participants auditory stimuli were irrelevant throughout the experiment.

**Results:**

ERPs showed a typical enhancement of early sensory specific components by intramodal spatial attention (visual group: visual P1 and N1; auditory group: auditory Nd). Crossmodal spatial attention effects included a modulation of the Nd to auditory standards in the visual group and a modulation of the P1 to visual novels and N1 for visual standards for the auditory group. Similar to previous studies crossmodal spatial attention effects on visual standard and novel stimuli also included a frontocentral positivity in the 200–400 ms range that was not seen for intramodal spatial visual attention suggesting involvement of later supramodal areas.

**Conclusion:**

These findings are consistent with an action of crossmodal spatial attention on early, sensory specific processing stages.

## Background

The function of selective attention is to facilitate the processing of stimuli, selected, for example, on the basis of their modality and location in space. Investigations on spatial attention have traditionally focused on selective processing within single sensory modalities. As many objects in everyday life (e.g., a car or an animal) provide us with information in more than one modality, it appears advantageous to process stimuli in other modalities coming from the attended region of space preferentially, as well. A case in point is the prototypical cocktail-party: In addition to selectively attend to the speaker's voice, it may be of additional value to attend to her/his lip movements. MacLeod und Summerfield [1], for example, have reported increases in intelligibility by 11 dB. Thus, crossmodal synergies are evident and the ventriloquist illusion powerfully illustrates that crossmodal integration is a robust phenomenon that might produce erroneous perceptions [2]. A number of behavioural studies (e.g., [3-5]) have found evidence for cross-modal links in endogenous (voluntary) spatial attention between vision, audition, and touch. In these studies, attention was (covertly) directed to the expected location of target stimuli within one (primary) modality. On some trials, stimuli of a different (secondary) modality were presented, that could appear on the same or on the other side to the expected location in the primary modality. The fact that superior performance was not only found for stimuli at the expected location in the primary modality but also in the secondary modality stimuli, suggests that the focus of attention within one modality influences the processing of information in other modalities. This has also been demonstrated by event-related potentials (ERPs, e.g., [6-10]). ERPs have the advantage that they can be recorded for stimuli that do not require a response and can thus demonstrate differential processing of secondary modality stimuli as a function of spatial attention in the primary modality. Intramodal spatial attention is associated with modality-specific signatures in the event-related brain potential (ERP). For example, attention to points in space in the visual modality gives rise to an enhancement of posterior P1 and N1 components to visual stimuli, with attention effects starting at around 80 ms [11-13]. The P1 is thought to arise from extrastriate occipital cortex [14], while the N1 has a generator in lateral occipito-temporal areas suggesting that the effects of attention on early stages of visual-perceptual processing are mediated by modality specific cortex [15]. In the auditory modality spatial attention is associated with an enhanced negativity for attended-location auditory stimuli starting around 100 ms and extending for several hundred milliseconds [16,17]. At least the early phase of this negative difference ('Nd') between the ERPs to attended and unattended stimuli likely emanates from auditory cortex [18].

Because both, auditory and visual attention effects can be linked to modality specific cortex, event-related brain potentials are very well suited to investigate whether the behavioral benefits of crossmodal spatial attention are similarly moderated by early sensory and perceptual processes or whether they arise at later stages.

With regard to the crossmodal interaction of endogenous spatial attention in vision and audition, two different ERP approaches can be differentiated. Eimer and Schröger [19] had participants either respond to visual deviants at attended locations and ignore all auditory stimuli or vice versa. Relevant locations were indicated by left- or right-pointing arrows at the beginning of each trial. In other words, transient attention shifts following the cue-stimulus were investigated in this study. When visual stimuli were relevant, enhanced visual P1 and N1 components were found for stimuli at the attended location. These effects were thus similar to previous intramodal visual attention studies using spatial cues [15]. However, when the auditory modality was attended, no modulation of the visual P1 amplitude was found. The following N1 component, on the other hand, did show a modulation as a function of direction of attention. This finding was viewed as evidence for crossmodal links in spatial attention from audition to vision modulating early stages of visual processing. Similar to previous unimodal auditory ERP experiments, auditory-spatial attention was reflected in an enhanced negativity for attended-location auditory stimuli in the Eimer and Schröger [19] study. A similar Nd effect was also present in the visual group. This was taken as evidence that visuospatial attention had an effect on early "sensory-specific" auditory processing. As crossmodal links in spatial attention affect sensory-specific ERP components arising from uni-modal sensory cortex, this indicates that such links modulate perceptual processes within modality-specific brain areas. In other words, spatial attention not only modulates processing in the primary, attended modality, but also influences modality-specific cortical processing in a currently irrelevant modality.

Using a blockwise manipulation of the direction of attention (left or right), Teder-Sälejärvi et al. [10], following earlier work [8], also found evidence for cross-modal effects on early processing stages moderated by sensory specific cortex. In this study, two groups of participants were tested, one only attending to auditory, the other attending exclusively to visual stimuli, thus ruling out a carry-over between experimental conditions. The stimulus series were identical for both groups and contained visual and auditory stimuli coming from left and right locations in fast succession. The visual group showed a typical enhancement of the posterior P1 and N1 components for attended vs. unattended visual stimuli, while in the auditory group, similar to Eimer and Schröger [19], no P1 modulation was seen. The posterior N1 was modulated by crossmodal spatial attention. Moreover, a positive shift (labeled "P204") was seen for frontal scalp sites for visual stimuli appearing at the location that was relevant for the auditory modality. For auditory stimuli a typical Nd effect was observed in the auditory group, which was also seen with the same scalp distribution albeit a smaller amplitude in the visual group.

Eimer [20] has interpreted these ERP results as evidence for symmetrical crossmodal links in spatial attention between vision and audition. Crossmodal spatial attention had an effect on early modality-specific components in the secondary modality, implying that it affects early sensory and perceptual processing stages. However, it cannot be overlooked that modulations of visual ERPs looked different if attention was directed within vision as compared to within audition. Specifically, no modulation of the visual P1 component was found if spatial attention was directed to auditory stimuli. Furthermore, an attention-dependent frontal positivity was elicited by visual stimuli in the auditory group of the Teder-Sälejärvi et al. [10] study, which typically is not present in visual attention studies.

The current experiment aimed to replicate and extend previous findings. A design very similar to Teder-Sälejärvi et al. [10] was employed. Instead of LEDs, video-monitors were used for stimulation to increase signal intensity in order to assess whether the missing modulation of the visual P1 in the crossmodal situation could have been due to the relatively low signal intensity in previous studies (see, for example, ref. [21] for the use of video-monitor instead of LEDs). Furthermore, infrequent visual and auditory novel stimuli (see below) were introduced into the stimulus series in addition to frequent standard and rare deviant stimuli. Numerous studies have shown that novel stimuli give rise to different ERP responses compared to rare stimuli that repeat within a sequence in the visual [22] and auditory [23] modality. Moreover, given the fact that novel stimuli are thought to have an alerting character and serve as a trigger for the orienting response [24] it was of interest to what extent these novel stimuli would be processed outside the spatial attentional focus in the relevant and irrelevant modality.

## Results

### Behavior

Reaction times did not differ between visual (414 ms, SD 32) and auditory (446 ms, SD 52) groups. In addition, intrachannel d' (possible false alarms: standard stimuli of the attended modality and of the attended location) was computed as a measure of perceptual sensitivity [25] and, again, no difference was found between visual (2.9, SD 0.25) and auditory (3.0, SD 0.60) groups. Thus, it can be concluded that the difficulty of auditory and visual attention tasks was roughly similar.

### Visual ERPs

**Figure 1:**
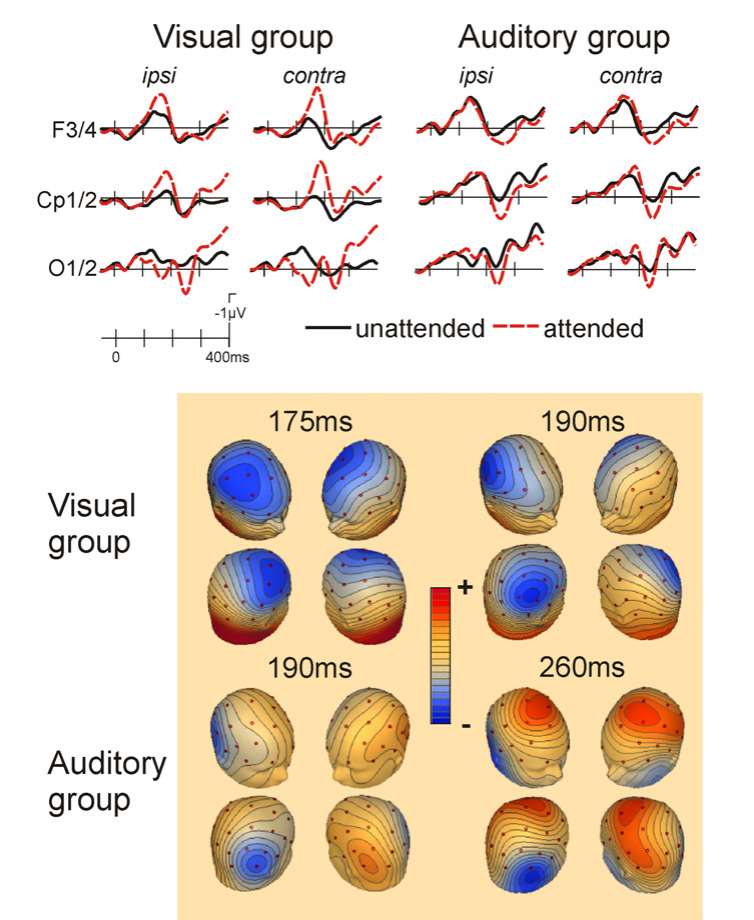
Grand average ERPs to visual standard stimuli for selected scalp sites and isovoltage scalp-maps (attended location ERPs minus unattended location ERPs; contralateral is right on these maps). The two left columns show the effect of spatial attention on the visual ERP in the "attend visual" group. A clear contralaterally distributed P1 component was seen at occipital electrodes, followed by an anterior N1 attention effect (seen at the F3/4 sites; upper left group of maps) and a somewhat later posterior N1 attention effect (seen at Cp1/2, upper right group of maps). Subsequent to the modulation of the P1, a second more ipsilateral positivity was present in the occipital channel with a peak latency of 250 ms. The right two columns depict the crossmodal effects of spatial auditory attention on visual ERPs in the "attend auditory" group. Here, no clear P1 modulation was present. Instead, only the second positivity at 260 ms was present in the occipital channels (see lower right group of maps). The frontal N1 effect seen in the visual group was greatly attenuated. Instead, visual standards at the attended location showed a positive displacement from 200 ms onwards at frontocentral sites. The posterior N1 clearly showed a modulation as a function of auditory spatial attention (see lower left group of maps). Maps depict the spline interpolated amplitudes at the specific time-points given.

The ERPs to the standard stimuli in the attend visual group show typical modulations as a function of spatial attention (figure 1, left side). At occipital and temporooccipital electrodes, the first attention sensitive component was an enhanced positivity (P1) in the 80 to 180 ms range. This attention effect showed a contralateral distribution as can be derived from the accompanying topographical maps. It was quantified by a mean amplitude measure (140–160 ms) on the posterior temporal and occipital electrodes, which yielded an attention by ipsi/contra interaction effect (F(1,10) = 10.07, p < 0.009). Post-hoc comparisons showed a significant effect for the contralateral (p < 0.001) but not for the ipsilateral (p = 0.15) electrodes. The next attention sensitive component was an enhanced frontocentral negativity in the 120–200 ms time range (frontal N1) for which a main effect of attention (mean amplitude 160–180 ms; electrodes F3/4, Fc1/2, C3/4; F(1,10) = 6.89, p < 0.025) and an attention x ipsi/contra interaction (F(1,10) = 9.83, p < 0.01) was seen. This interaction was followed up by post-hoc comparisons which revealed significant attention effects for both, contralateral (p < 0.001) and ipsilateral (p < 0.05) electrodes.

At more posterior sites, a second, slightly later, negativity (peak latency 180 ms) was observed that had a contralateral distribution and was larger for the attended stimuli. This was reflected by a main effect of attention (mean amplitude 180–200 ms, CP1/2, CP5/6 and P3/4 sites, F(1,10) = 8.31, p < 0.02) as well as an attention x ipsi/contra interaction (F(1,10) = 11.98, p < 0.006). Post-hoc comparisons revealed an attention effect for both, contralateral (p < 0.002) and ipsilateral (p < 0.04) electrodes.

For the attend auditory group, a modulation of the visual ERPs as a function of the direction of auditory spatial attention was evident (figure 1, right). No reliable modulation of the P1 component was observed. The first attention effect was seen for the posterior N1 (Cp1/2, P3/4, P7/8, O1/2, 180–200 ms, attention x ipsi/contra F(1,10) = 8.19, p < 0.02, post-hoc comparisons showed an attention effect contralateral, p < 0.025, but not ipsilateral). This was followed by a more positive peak, which was larger for the visual stimuli appearing at the attended side (same electrodes, 230–250 ms; attention main effect: F(1,10) = 12.64, p < 0.005, attention x ipsi/contra F(1,10) = 13.94, p < 0.004; post-hoc comparisons showed an attention effect for both, contralateral, p < 0.05, and ipsilateral, p < 0.002, sites). At frontal sites, a positive shift was seen for the stimuli occurring on the attended side (F3/4, C3/4, Fc1/2, 250–350 ms; attention main effect F(1,10) = 7.90, p < 0.02).

**Figure 2:**
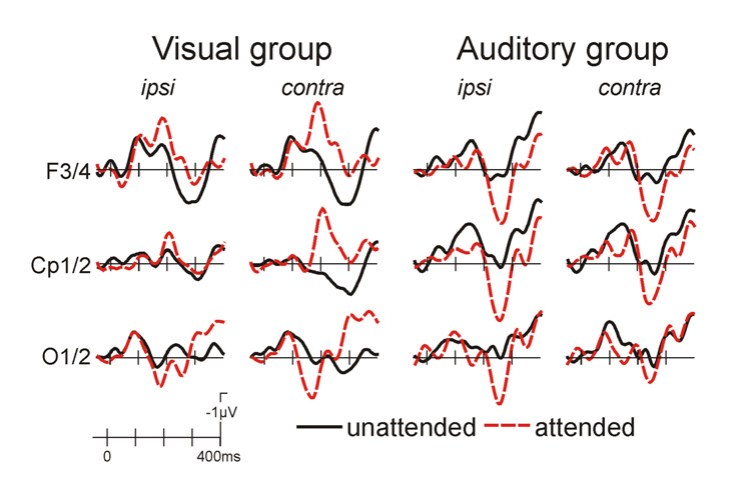
Grand average ERPs for the visual novel stimuli. At occipital sites a clear contralateral P1 modulation was found in the visual as well as for the auditory group. In the visual group, an enhancement of the frontal as well as parietal N1 was observed. By contrast, the auditory group showed a more positive waveform for the attended stimuli.

For the visual novel stimuli a typical attention dependent modulation was observed in the visual group (fig. 2, left), which included a mainly contralateral enhancement of the posterior P1 (P7/8, O1/2, 140–160 ms; attention x ipsi/contra F(1,10) = 15.94, p < 0.003; post-hoc comparisons, attention effect at contralateral, p < 0.001, and ipsilateral, p < 0.04, electrodes), parietal N1 (Cp1/2, P3/4, 180–200 ms; attention x ipsi/contra F(1,10) = 13.58, p < 0.004; post-hoc comparison, attention effect at contalateral sites, only, p < 0.005) and frontal N1 (F3/4, C3/4, Fc1/2; 180–200 ms, attention x ipsi/contra F(1,10) = 39.90, p < 0.0001, post-hoc comparisons showed attention effect for contralateral, p < 0.001, and ipsilateral, p < 0.01, sites).

In the auditory group, novels were similarly associated with a modulation of the posterior P1 (P7/8, O1/2, 140–160 ms; attention F(1,10) = 5.42, p < 0.05; attention x ipsi/contra F(1,10) = 4.11, p = 0.07), which is in contrast to the standard stimuli. Moreover, an enhancement of a second posterior positive peak was observed (P7/8, O1/O2, 230–250 ms; attention F(1,10) = 11.48, p < 0.007). At frontal sites, a similar positivity was observed as for the standard stimuli (F3/4,Fc1/2,C3/4, 220–280 ms; attention F(1,10) = 6.36, p < 0.03).

**Figure 3:**
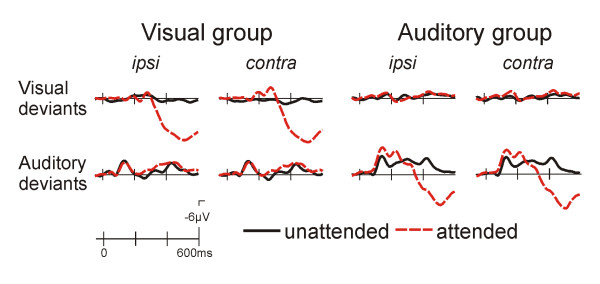
Grand average ERPs to the deviant stimuli from the Cp1/2 site. It is apparent that a P3 response is only observed for attended location stimuli in the attended modality. This is preceded by an contrallateral negativity for visual deviants in the visual group and an N2b response for auditory deviants in the auditory group.

The ERPs to visual deviants can be derived from figure 3. An N2/P3 complex was seen for the attended deviants (targets) in the visual group, which was not present in the auditory group. The P3 was quantified by a 400–600 ms mean amplitude measure (electrode sites: Cp1/2, P3/4; visual group: attention F(1,10) = 45.3, p < 0.001; auditory group: n.s.).

### Auditory ERPs

**Figure 4:**
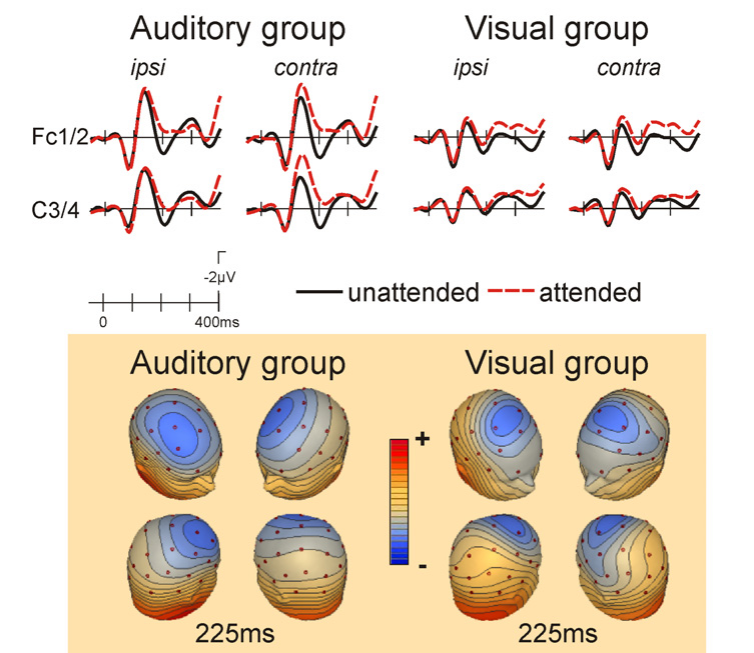
ERPs to auditory standard stimuli. In the "attend auditory" group a typical frontocentral enhanced negativity was seen for attended location stimuli. A similar effect was also seen in the attend visual group. The scalp topography of the attended location minus unattended location difference maps is very similar for the attend auditory and the attend visual groups.

In the auditory group a typical modulation of the ERP to standard stimuli was seen as a function of the direction of auditory attention (fig. 4, left). The ERP to the attended stimuli was more negative than that to the unattended stimuli. This Nd effect was maximal fronto-centrally and was significant in an early (F3/4, C3/4, Fc1/2; 100–250 ms; attention F(1,10) = 8.85, p < 0.02, attention x ipsi/contra F(1,10) = 13.24, p < 0.005) and a later time window (same electrodes, 300–650 ms; attention x ipsi/contra F(1,10) = 7.56, p < 0.02).

A similar but smaller modulation was found in the visual group (fig. 4, right; F3/4, C3/4, Fc1/2; 100–250 ms: attention x ipsi/contra F(1,10) = 6.16, p < 0.04; 300–650 ms: attention F(1,10) = 6.85, p < 0.03).

**Figure 5:**
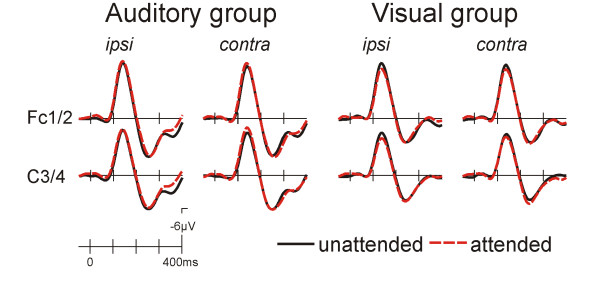
ERPs to auditory novel stimuli. In the "attend auditory" group a large frontocentral negativity with a peak latency of 140 ms was present that did not show a modulation as a function of attention. A very similar, only slightly smaller ERP response to auditory novel stimuli was seen in the "attend visual" group. No influence of the direction of visual attention was found

For the auditory novel stimuli (fig. 5), a large frontocentral negativity peaking at 140 ms followed by a positive peak at 240 ms was observed. This positive deflection represents the P3a component. An attentional modulation of the ERP was neither seen in the auditory nor in the visual group. In fact, except for the fact that the P3a was somewhat smaller in the visual group the ERPs from both groups were very similar. The smaller P3a was corroborated by a between groups ANOVA (Fz,Cz,Pz, 200–400 ms: group F(1,20) = 6.40, p < 0.02).

The attended auditory deviants were associated with an N2b/P3 complex in the auditory group, while no P3 modulation is present in the visual group (fig. 3). Correspondingly, the P3, quantified by a 400–600 ms mean amplitude measure (electrode sites: Cp1/2, P3/4) was significant in the auditory group only (attention main effect; F(1,10) = 31.8, p < 0.001; visual group: n.s.).

## Discussion

The present study replicates and extends previous results (e.g. [8,10,19,20]): Paying attention to a particular point in space in one modality, visual or auditory, led to modulations of early sensory-specific ERP components to stimuli in the other modality. Thus, it can be concluded that the processing of unattended modality stimuli occurring at attended locations is facilitated at an early, sensory level.

With regard to visual ERPs, previous studies using sustained attention tasks similar to one used in the current experiment have not found a modulation of the posterior P1 component when attention was directed towards auditory stimuli. The earliest signs of crossmodal spatial attention effects on visual stimuli were obtained only on the subsequent posterior N1 component. For the standard stimuli in the present experiment, a similar situation was found, i.e., no enhancement of the visual P1 in the auditory group. The P1 has been localized to ventro-lateral extrastriate cortex [14], and is the earliest electrophysiological sign of visuo-spatial attention, while the N1 component occurs some 50 ms later. On the basis of a differential sensitivity to task manipulations it has been suggested that the P1 component might index facilitation of early sensory processing for items presented to a location where attention is already focused, while the N1 has been proposed to reflect the orienting of attention to a task-relevant stimulus [12].

The question therefore arises whether the P1/N1 dissociation found for standard visual stimuli in the auditory group of the present study as well as previous studies [10,19] (a) indicates that crossmodal spatial attention acts only at later (i.e. post P1) stages of sensory processing, (b) reflects the fact that visual stimuli at the attended location lead to an orienting of attention towards these stimuli (c.f. ref [12]), or (c) is simply due to the fact that the visual stimuli used were of insufficient intensity to elicit a P1 modulation.

The fact that in the current study visual novel stimuli were associated with a clear attention effect for the P1 in the auditory group strongly argues for the latter possibility. Please recall, that visual novel stimuli were of created from colored checkerboard patterns. The early (80 ms, c.f. figure 2) crossmodal spatial attention effect therefore indicates that crossmodal spatial attention acts at the same level in the visual system as intramodal visual spatial attention. Why, then, is there an early visual spatial attention effect in the attend visual group for the standard stimuli? Previous ERP-studies [26] have shown that ERPs in the unattended modality are generally attenuated ("intermodal attention effect"). This implies that for standard stimuli in the present as well as in previous studies [10,19] an P1 modulation might have been masked by the "intermodal" effect.

The attention effects for the auditory standard stimuli were very similar in the attend auditory and the attend visual groups: In both cases an Nd effect was present, thus replicating earlier studies [10]. It can thus be concluded that spatial attention to stimuli in one modality therefore facilitates processing of stimuli in the other modality that occur at the same location at the level of modality-specific cortex in a largely symmetrical fashion: visual attention facilitates the processing of attended location auditory stimuli and vice versa. Such facilitated processing is the likely cause of crossmodal perceptual cueing effects [3,4,27].

While the present results provide evidence for similarities in the mechanisms of intramodal spatial attention and crossmodal spatial attention on visual stimuli (posterior P1 and N1 enhancement), the modulations of visual stimuli were very different in the visual and auditory groups from 200 ms onwards. In particular, the frontal N1 effect seen in the visual group was absent in the auditory group. This group rather showed a frontal positivity for the attended location stimuli, which was similar to the "P204" of Teder-Sälejärvi et al. [10]. These authors speculated that this qualitative difference might indicate the participation of additional, polysensory cortical areas in the crossmodal situation. Unfortunately, the signal to noise ratio of the current data does not warrant source localization of this effect but scalp-topographic mapping clearly indicates a fronto-central distribution of this effect.

A second aim of the current study was to investigate to what extent the processing of highly deviant rare novel stimuli would depend on spatial attention when the modality of the novel stimuli was relevant or when it was irrelevant. The results were very clear: For novel auditory stimuli, no effects of spatial attention or attended modality were found for the first 200 ms post-stimulus, indicating a highly automatic and attention-independent processing of these stimuli. By contrast, the ERPs to the visual novel stimuli were influenced by the direction of spatial attention. This was not only the case when the visual modality was relevant but also when the auditory stimuli were relevant.

This different treatment of novel stimuli in the two modalities likely results from the fact that spatial location in vision is available automatically by virtue of the retinotopic organization of this system, while location must be actively computed from multiple cues in audition.

## Conclusion

Attention to points in space in one modality boosts the neural responses to task-irrelevant stimuli from the same location in another modality. Crossmodal spatial attention effects are implemented by modulating the activity in unimodal cortical areas. Visual and auditory modalities behave differently with regard to novel stimuli: While the processing of visual novel stimuli requires spatial attention regardless of whether the visual modality is relevant or not, auditory novel stimuli are processed independent of allocation of spatial attention.

## Methods

All procedures were approved prior to the study by the ethics committee of the University of Magdeburg, which ensured compliance with the Helsinki Declaration.

### Participants

Two groups of healthy young, right-handed student volunteers gave informed consent to participate in a single experimental session. The "attend auditory" group (n = 11, 7 women, mean age 24.6 years) was instructed to attend exclusively to auditory stimuli, while the "attend visual" group (n = 11, 3 women, mean age 25.6 years) had to pay attention to the visual stimuli, only. Participants were either paid or received course credit.

### Stimuli and procedure

Rapid stimulus sequences comprising auditory and visual stimuli were presented using two speakers and computer monitors located 30 degrees left and right of a central fixation point. The speakers were standard broadband models intended for use in car-HiFi systems and had a diameter of 10 cm. They were placed on top of each monitor about 10 cm above the location of the visual stimuli. Tone stimuli consisted of frequent (p = 0.4 with regard to the combined audio-visual sequence) computer-generated 800 Hz "standard" tones of 60 ms duration (10 ms rise and fall times) and 900 Hz "deviant" tones of the same duration (p = 0.05). Moreover, novel stimuli of 60 ms duration (p = 0.05) that were generated by simultaneously presenting three sinusoidal tones (frequency range 1.8 to 5.5 kHz, frequencies picked at random) were inserted to the stimulus sequence. None of the novel tones was repeated during the experiment. In a pilot study, novel stimulus intensity was adjusted to yield the same subjective loudness as judged by five further young volunteers who did not participate in the main experiment. The tone sequences were presented in random order to the left and right speaker.

In the visual modality, a white rectangle (3.9 by 4.6 degrees visual angle, luminance 71 cd/m^2^) of 100 ms duration served as the "standard" stimulus (p = 0.4), whereas visual deviants (p = 0.05) included a smaller black rectangle (0.8 by 0.9 degree visual angle, luminance 67 cd/m^2^) at the center of the white rectangle. Visual novel stimuli (p = 0.05, 50 ms duration) consisted of a square (3.9 by 3.9 degrees visual angle) that was made up from four small squares, two of one color and two of another (checkerboard stimulus; luminance between 28 and 54 cd/m^2^). Novel stimuli were composed of the random combination of the colors red, blue, pink and green.

The combined auditory and visual stimulus sequence was presented with a mean ISI of 150 ms. In any given experimental run (180 seconds duration), "attend auditory" participants had to attend to one location (left or right) in order to detect auditory deviant stimuli at the designated position, while ignoring all visual stimuli, and the auditory stimuli at the unattended location. The participant was required to respond to target deviant tones by pressing a button located beneath the right index finger as fast as possible. Thus, participants in the attend auditory group did not have to respond to visual stimuli at any time. In fact, the visual sequence did not contain any important information for the participants. Correspondingly, the "attend visual" group was asked to respond (by button press) to visual deviant stimuli at the designated positions while ignoring the auditory stimuli. Novel stimuli of either modality were irrelevant. Participants in both groups were required to fixate a cross placed directly in front of them at a distance of 1.3 m. A total of 20 runs (10 attend left, 10 attend right) were administered in a single session. This yielded 8600 standard stimuli, 1000 target and 1000 novel stimuli per modality.

### Recording and analysis

The EEG was recorded from the scalp using tin electrodes mounted in an electrode cap located at 29 positions including all standard positions of the international 10/20 system. The horizontal EOG was recorded using a bipolar montage between two electrodes mounted on the left and right external canthus. The vertical EOG was recorded between electrodes placed below and above the right eye to allow off-line rejection of ocular artifacts. The EEG was amplified (time-constant 10 seconds, low pass filters 70 Hz) and digitized on-line with 4 ms resolution. EEG-signals were re-referenced off-line to the mean of the activity at the two mastoid processes. Artifact rejection for eye-blinks and amplifier blocking was performed by a special purpose program using individualized amplitude criteria that were determined by measuring the typical amplitudes of each participant's blink artifacts (thresholds varied between 60 and 90 mV). This resulted in a loss of maximally 25 % of the trials. ERPs were obtained for epochs of 1024 ms including a 100 ms interval before the onset of the stimulus used for base-lining. The ERPs were averaged separately for group (attend auditory/attend visual), attention condition (attended/unattended), location (left/right), stimulus class (auditory/visual) and stimulus type (standard/deviant/novel). Initial statistical analyses showed that there was no significant main effect for stimulus location (left/right). Thus, ERPs to left and right stimuli were collapsed for further analysis to increase the signal to noise ratio in such a way that contralateral (e.g. F3 for right stimuli averaged with F4 for left stimuli) and ipsilateral electrodes (e.g. F4 for right stimuli and F3 for left stimuli) were obtained. ERPs were quantified by mean amplitude measures and the resulting data were subjected to repeated measures analyses of variance (ANOVAs) for each group separately. Measurements were restricted to the fronto-central and temporo-occipital region where auditory and visual attention and effects are largest. Specific electrodes used are given in the result section. Electrodes were grouped according to whether or not they were ipsi- or contralateral to the spatial location of the eliciting stimuli, thus yielding "hemisphere" (levels: ipsi, contra) and "site" factor (2 or 3 levels depending on the electrode set entered into the statistics). Attention main effects and attention by contra/ipsi interactions are reported in the result section. For display, ERPs were digitally low-pass filtered (13 Hz half amplitude cutoff).

## Abbreviations

Nd: negative displacement (for auditory attention effect)

ERP: event-related potential

## Authors' contributions

WN co-designed the study, analysed the experiments, and wrote the first draft of the manuscript, KE performed part of the experiments and performed some statistical analyses, TFM co-designed the study and wrote the final draft of the manuscript. All authors read and approved the final manuscript.
